# Incidence, National Trend, and Outcome of Nontraumatic Subarachnoid Haemorrhage in Taiwan: Initial Lower Mortality, Poor Long-Term Outcome

**DOI:** 10.1155/2014/274572

**Published:** 2014-03-31

**Authors:** Hsing-Lin Lin, Kwan-Ming Soo, Chao-Wen Chen, Yen-Ko Lin, Tsung-Ying Lin, Liang-Chi Kuo, Wei-Che Lee, Shiuh-Lin Huang

**Affiliations:** ^1^Division of Trauma, Department of Surgery, Kaohsiung Medical University Hospital, 100 Tzyou 1st Road, Kaohsiung 807, Taiwan; ^2^Department of Emergency Medicine, Kaohsiung Medical University Hospital, Kaohsiung Medical University, Kaohsiung, Taiwan; ^3^Faculty of Medicine, College of Medicine, Kaohsiung Medical University, Kaohsiung, Taiwan; ^4^Department of Surgery, Kaohsiung Medical University Hospital, 100 Tzyou 1st Road, Kaohsiung 807, Taiwan; ^5^Division of Neurosurgery, Department of Surgery, Kaohsiung Medical University Hospital, Kaohsiung Medical University, Kaohsiung, Taiwan

## Abstract

To investigate the longitudinal trend of nontraumatic subarachnoid haemorrhage (SAH), we analyzed the annual population-based incidence and mortality rate of nontraumatic subarachnoid hemorrhage in Taiwan. Logistic regression was used to identify independent predictors of mortality. The average incidence rate (IR) of nontraumatic SAH was 6.25 ± 0.88 per 100,000 per year. The prevalence of female patients was higher than in the male population (54.5% versus 45.5%). The average age of these patients was 55.78 ± 17.09 and females were older than males (58.50 ± 15.9 versus 52.45 ± 18.50, *P* < 0.001). Of these patients, 97.6% (611/626) were treated with surgical intervention with clipping procedure and 2.9% (18/626) with coiling. Total mortality of these patients was 13.4% (84/626). In adjusted analysis, age (odds ratio [OR], 0.97; 95% confidence interval [CI], 0.98-0.98; *P* < 0.001) and Charlson comorbidity index (OR, 0.709; 95% CI, 0.57–0.88; *P* = 0.002) remained independent predictors of the mortality. Patients with nontraumatic SAH had a much higher prevalence in older age groups and in females than in the general population. Patients with old age and more comorbidity have higher mortality. Aggressive management of patients might reduce the initial mortality; however, patient outcome still remains poor.

## 1. Introduction

Nontraumatic subarachnoid hemorrhage (SAH) is a critical and serious disease. After rupture, patients usually suffer severe headache and consequent loss of consciousness. According to previous studies, countries with high incidence of around 20 per 100,000 person years (PY), such as Finland and Japan, and countries with low incidence of approximately 5–10 per 100,000 PY in The Netherlands [1, 2]. Significant variation in incidence rates between countries are reported for nontraumatic SAH [3]. In the studies from Europe, there might be existing regional differences [4–7]. Seasonal variation, diurnal, and daily factors were also found to be associated with the incidence of nontraumatic subarachnoid hemorrhage [8, 9].

Most patients found to have that this disease occurred suddenly without portend. In previous studies, nontraumatic SAH was found to affect more females and the older population [2, 6, 7, 10]. Global mortality ranges from 32 to 67% [11] with around 20% of SAH patients dying before arriving at the hospital [12]. Most of the nontraumatic SAH needed emergency intervention, such as aneurysm clipping or angioembolization with coil [13]. Endovascular coiling as compared to neurosurgical clipping had better general clinical outcomes [14, 15]. Hypertension, smoking, high alcohol intake, and extreme physical exertion were considered as risk factors of SAH [16, 17].

Many developed countries are facing the challenge of an aging population. With an increasingly aging population, the occurrence of nontraumatic SAH will predictably rapidly increase and will represent a major and growing health care problem. Thus, with a very large variation in incidence of nontraumatic subarachnoid hemorrhage in this region and many countries lacking comprehensive data and available reports, we conducted this study to investigate the trend of epidemiology and outcome of nontraumatic SAH from 2000 to 2009 in Taiwan and compared our result with other countries.

## 2. Methods

### 2.1. Database

Taiwan implemented an NHI program in March 1995, offering a comprehensive, unified, and universal health insurance program to all citizens. We conducted a nationwide survey of nontraumatic SAH in Taiwan from 2000 to 2009 based on the National Health Insurance Research Database. This cohort dataset comprised 1,000,000 randomly sampled beneficiaries still enrolled in the NHI program during 2000 and collected all records on these individuals from 1995 to 2009 containing basic demographic information on insured residents (sex, age, region, and so on), along with medical records (including inpatient and ambulatory visits). Healthcare facilities contracted under the NHI provide the insured of the NHI with inpatient care, ambulatory care, dental care, and prescription drugs. The claims data of the NHI are routinely monitored by the Bureau of the NHI for their accuracy and completeness. According to the Taiwan National Health Research Institute, there were no statistically significant differences in age, gender, or health care costs between the sample group and all beneficiaries under the NHI program. The study protocol was approved by the Institutional Review Board of Kaohsiung Medical University.

### 2.2. Study Sample

The International Classification of Disease, Ninth Revision (ICD-9) to define nontraumatic SAH was used to claim the data. From the NHI inpatient database, we identified people newly diagnosed with ICD-9-CM codes from 2000 to 2009 with codes: 430.0 (ruptured aneurysm), 430 (subarachnoid hemorrhage), and 437.3 (unruptured aneurysm) of brain aneurysm and ICD-9 codes. The treatment codes with 39.51 (clipping of aneurysm), 39.79, 39.72, 39.25 (coiling), 38.31 (suture of artery), 39.52 (other repair of aneurysm), 38.81, and 38.82 (other surgical occlusion of intracranial vessels) were also used for inclusion criteria. The exclusion criteria included 748.8 (arteriovenous malformation). Because of this financial incentive, almost everyone with aneurysm in Taiwan is included in the health system; thus, the identification of brain aneurysm from the database should be nearly 100%. In addition, all the diagnoses and management of brain aneurysm were by neurosurgeons in Taiwan; thus, the brain aneurysm cases identified from the dataset should be accurate. Mortality rate was defined within 30 days of hospital admission. Because Taiwan's NHI is the only payer health program, there is no other reason for being withdrawn from the NHI coverage within 30 days of hospital admission expect for patient mortality. Overall mortality is estimated if patients do not expire over more than 3 months but expire during the study period. The coding algorithm reported by Deyo et al. was used to identify 17 prognostic comorbidity conditions and calculate the Charlson comorbidity index (CCI) according to specific weights assigned to each condition [18, 19].

### 2.3. Statistical Analysis

Mean, median, and interquartile range (IQR) were generated for continuously coded variables. Frequencies and proportions were generated for categorical variables. The categorical data between the two groups were compared with chi-square test and Fisher exact test as appropriate. Comparisons between continuous variables were done by Student's *t-*test. One-way ANOVA rated the difference of age between years and disease occurrences between seasons. Subsequently, binary unconditional logistic regression models were fitted to test the effect of age, gender, and CCI on the mortality. Statistical significance was inferred at a 2-sided *P* value of <0.05. All statistical analyses were carried out using the Statistical Package for Social Science, version 19.0 (SPSS, Chicago, IL).

## 3. Results

**Figure 1:**
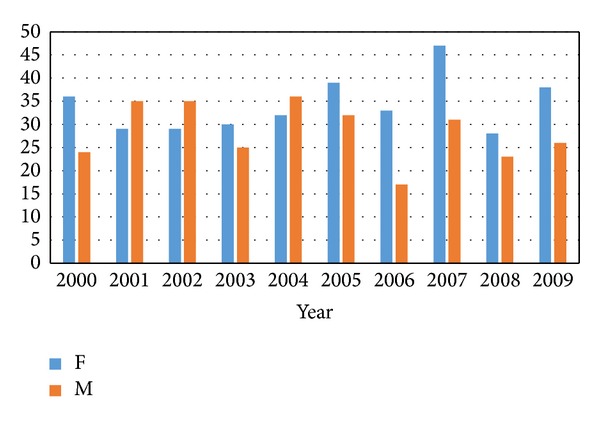
The percentage of a total 625 patients from 2000 to 2009 with no trend of increasing or decreasing patient number.

Overall, 627 patients with nontraumatic SAH were identified in the database. One patient with missed data of gender was excluded. The average incidence rate (IR) of nontraumatic SAH was 62.5 ± 8.82 per 1,000,000 per year. There was no significant association between gender and years (chi-square test; *P* = 0.280) (Figure 1). The prevalence of female patients was higher than that of males (54.5% versus 45.5%).

**Figure 2:**
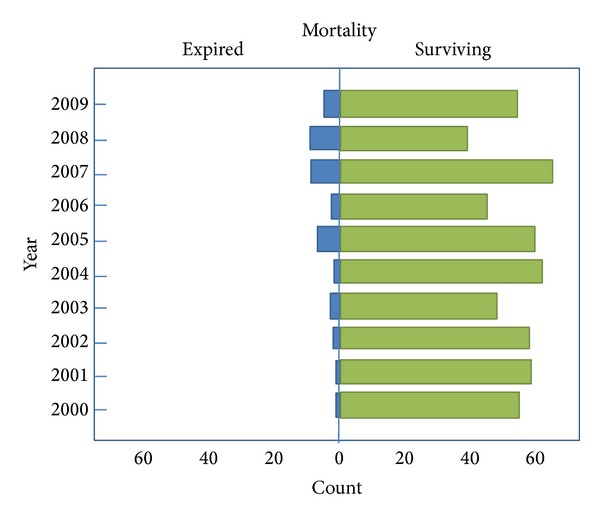
The trend of mortality from 2001 to 2009.

Overall, total mortality of these patients was 13.4% (84/626). The mortality of each year showed differences (*P* = 0.06). There was a trend of increasing mortality from 2000 to 2009 (Figure 2). However, there was no difference of mortality between gender (females versus males: 13.7% [47/344] versus 12.8% [37/288]; *P* = 0.885) and performing of tracheostomy (Yes versus No 7.8% [4/51] versus 6.4% [37/574]; *P* = 0.699).

**Figure 3:**
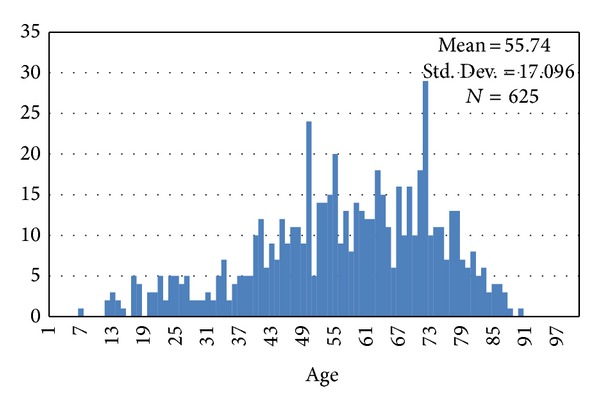
The distribution of age in patients having nontraumatic subarachnoid haemorrhage.

The average age of these patients was 55.78 ± 17.09 (Figure 3). Females found to have the disease were older than males similarly affected (*P* < 0.001). Nonsurviving patients were older on average than those surviving (61.95 ± 17.23 versus 55.30 ± 17.02; *P* = 0.016). The distribution of patient's age had no difference from 2002 to 2009 (one-way ANOVA; *P* = 0.209). In adjusted analysis, age (odds ratio [OR], 0.97; 95% confidence interval [CI], 0.98-0.98; *P* < 0.001) and Charlson comorbidity index (OR, 0.709; 95% CI, 0.57–0.88; *P* = 0.002) remained as independent predictors of mortality.

There were no differences of disease occurrence between seasons (one-way ANOVA; *P* = 0.661). Of these patients, 97.6% (611/626) were treated with surgical intervention with clipping procedure and 2.9% (18/626) with coiling. For treatment location, 70.4% (441/626) patients were treated at medical centers; 27.3% (171/626) patients were treated at regional hospitals; and 2.2% (14/626) patients were treated at district hospitals. During the study period, the overall survival rate of these patients was 71.9% (455/626).

## 4. Discussion

In this study, we found that patients with nontraumatic SAH had a much higher prevalence in older age groups and in females than in the general population. Toward the end of the study period, similar overall outcomes were observed in the trend of incidence and mortality. When compared to other countries, incidence in Taiwan was similar to other countries but the initial mortality rate was lower.

The 30-day case-fatality rate of the hospitalized nontraumatic SAH was high with range from 30% to 34.7% [1, 7, 20]. In the previous studies, the median case fatality rate in Europe was above 40%; in Asia it was about 35.8% except for Japan at 26.7%; in South America and the Caribbean it was 32.5%; in Australia and New Zealand it was 41.7%; and in the USA it was 32.2% with publications between 1965 and July 2007, and the period of survey was later than 1960 [21]. In our study, we found that the mortality rate was lower than other countries (13.4%). However, operative rate was higher in our study group. The medical policy in Taiwan allows most patients to receive intensive care with surgical intervention without worrying about the medical expense. The higher surgical rate is because the financial policies do not pay for coiling; this impacts the treatment decision for patients during the study period. With aggressive medical treatments, mortality may be reduced and become lower than in other countries. In addition, 97.4% patients of these patients were treated at medical centers and regional hospitals. Surgical intervention of brain aneurysm requires a skillful neurosurgeon and these facilities have staff capable of managing these patients; therefore, the lower mortality may result from easy accessibility of medical resources.

There are few studies of the incidence of nontraumatic aneurysm in Asia. Although the aggressive treatment of the aneurysm may result in lower mortality within 30 days; however, the overall mortality reaches 30% eventually. The health system in Taiwan provides relatively better financial support and patients can receive aggressive treatment including 97.6% of patients being treated with surgical intervention without consideration of medical expense, which may improve the short-term survival rate. In addition, most of the EMTs can deliver a critical patient to a hospital within 30 minutes, and this can decrease secondary brain injury after a stroke episode. Therefore, the health providing systems may rather influence the short-term outcome of patient mortality. Similar results of lower mortality can be found in the studies from Japan, where the medical system is similar to Taiwan. Our study showed a trend of increase in incidence and mortality rates of aneurysmal SAH in Taiwan. After 2000, the Hospice Palliative Act was implemented. Some of these patients might give up after finding poor outcome [22], which might increase the mortality within 30 days after the implementation of the law.

Age was found to be a predictor of poor outcome [7]. In this study, we found that age and Charlson comorbidity index remained independent predictors of mortality. Older people have more comorbidity than do young people. There was no statistical difference of mortality between seasons, which may result from the tropical location of Taiwan where temperature change is not obvious related to seasonal change. Females are prone to have nontraumatic SAH; however, their average age was older than that for males. The protective effect may result from hormonal influence before menopause [23, 24].

The strengths of this study include its nationwide population and the large number of patients included in the analysis. Based on observational data, the results of our study should be interpreted in the light of potential limitations, such as selection bias and information bias. First, in common with other studies using administrative databases, no information on nontraumatic SAH severity was available for risk adjustment. Patients who died before reaching hospital were not included in the NHI estimate, and it is highly likely that they were also missed in the NHI database due to lack of proper diagnosis and specialist information. This means that the true incidence is potentially higher than in our study. Previous studies show that the percentage of persons dying before reaching hospitals was between 11 and 13% [7, 12]. Second, the selection of variables was limited because the data source was a secondary one. Because of lack of information on processes of care, we could not identify the unmeasured variables that might explain the differences in nontraumatic SAH mortality. Third, nontraumatic SAH diagnoses are based on hospitals' claims, so the accuracy of nontraumatic SAH coding could be questioned. However, it must be noted that the NHI regularly and randomly samples a percentage of cases from hospitals to verify the validity of diagnosis and quality of care through chart reviews using touring professional teams, and all the diagnosis of the codes were by the neurosurgeons. We think this error is limited in the NHI database as we applied a very sensitive search on codes. Further prospective study focused on the predictor factors of patients deemed to be prone to mortality may decrease futile treatment. If further advance of intervention such as angioembolization can provide better outcome, aggressive treatment might provide more opportunity for patient survival.

## 5. Conclusion

Patients with old age and more comorbidity have higher mortality. In Taiwan, the initial mortality rate of nontraumatic aneurysm is lower as the health provision system might improve incidence of short-term mortality; however, the overall mortality was found to be similar to other countries. Aggressive management of patients might reduce the initial mortality; however, patient outcome still remains poor.
